# Estrogen levels in young women with hormone receptor-positive breast cancer on ovarian function suppression therapy

**DOI:** 10.1038/s41523-024-00680-0

**Published:** 2024-08-01

**Authors:** Megan E. Tesch, Yue Zheng, Shoshana M. Rosenberg, Philip D. Poorvu, Kathryn J. Ruddy, Rulla Tamimi, Lidia Schapira, Jeffrey Peppercorn, Virginia Borges, Steven E. Come, Craig Snow, Shalender Bhasin, Ann H. Partridge

**Affiliations:** 1 Department of Medical Oncology, Dana-Farber Cancer Institute, Boston, MA USA; 2 Breast Oncology Program, Dana-Farber Brigham Cancer Center, Boston, MA USA; 3 Harvard Medical School, Boston, MA USA; 4 Department of Data Science, Dana-Farber Cancer Institute, Boston, MA USA; 5 Weill Cornell Medicine, New York, NY USA; 6 Mayo Clinic, Rochester, MN USA; 7 Stanford University, Stanford, CA USA; 8 Massachusetts General Hospital, Boston, MA USA; 9 University of Colorado Cancer Center, Denver, CO USA; 10 Beth Israel Deaconess Medical Center, Boston, MA USA; 11 Brigham and Women’s Hospital, Boston, MA USA

**Keywords:** Breast cancer, Breast cancer, Breast cancer

## Abstract

Ovarian function suppression (OFS) benefits young women with hormone receptor (HR)-positive breast cancer but they are at risk for ovarian function breakthrough. We assessed endocrine effects of gonadotropin-releasing hormone agonist (GnRHa) treatment in a prospective cohort of patients aged ≤ 40 years with HR-positive breast cancer. Plasma estradiol (E2), estrone, and follicule-stimulating hormone (FSH) levels were measured from blood samples drawn 1 and 4 years after diagnosis. Patient characteristics, invasive breast cancer-free survival (iBCFS), and overall survival (OS) were compared between those with and without E2 > 2.72 pg/mL during GnRHa treatment. Among eligible patients, 54.7% (46/84) and 60% (15/25) had E2 > 2.72 pg/mL at 1 and 4 years, respectively. Factors associated with E2 > 2.72 pg/mL at 1 year were no prior chemotherapy (*P* = 0.045) and tamoxifen use (*P* = 0.009). After a median follow-up of 7 years, among patients with stage I-III breast cancer (*N* = 74), iBCFS events were seen in 6 (8.1%) with E2 > 2.72 pg/mL and 5 (6.8%) with E2 ≤ 2.72 pg/mL (*P* = 0.893). Among patients with de novo metastatic breast cancer (*N* = 12), 6 (50%) with E2 > 2.72 pg/mL and 3 (25%) with E2 ≤ 2.72 pg/mL died during follow-up (*P* = 0.052). Larger studies exploring the clinical implications of incomplete E2 suppression by GnRHa are needed to ensure optimal OFS treatment strategies are being employed for this population.

## Introduction

Large, randomized trials have demonstrated that the addition of ovarian function suppression (OFS) to adjuvant oral endocrine therapy improves survival outcomes for premenopausal women with early-stage, hormone receptor (HR)-positive breast cancer^1–3^. In the advanced setting, OFS has been a therapeutic strategy for over a century^4^, and remains an essential component of treatment for premenopausal, endocrine-sensitive, metastatic breast cancer^5^. Although oophorectomy was once the preferred method for OFS, gonadotropin-releasing hormone agonists (GnRHa) are now favored for most patients given their less invasive and reversible suppression of ovarian function^6^.

In the Suppression of Ovarian Function Trial (SOFT), the greatest benefits from OFS were seen in patients aged <35 years^2^. Yet, younger women also face greater risk for incomplete ovarian suppression (so called “breakthrough”) on GnRHa therapy. In the SOFT-EST analysis of the SOFT trial, OFS breakthrough was most frequently observed in patients aged < 35 years, with four of eight within this age group having premenopausal estradiol (E2) levels on triptorelin plus exemestane^7^. Suboptimal E2 suppression by GnRHa is of particular concern for premenopausal patients on concurrent aromatase inhibitor (AI), given the hypothalamic-pituitary-ovarian axis is activated in response to the decrease in circulating estrogen levels by AI^8^. This activation leads to an increase in gonadotropin secretion and consequent enhanced ovarian estrogen synthesis, such that AI may even promote ovarian functioning in premenopausal breast cancer patients^9,10^. In light of this, it has been recommended to avoid AI use when E2 levels are above 10 pmol/L (2.72 pg/mL), or not low enough for an AI to exert full effectiveness^11^.

Standard assays in clinical practice lack the sensitivity and specificity to detect the low estrogen levels sought during combined GnRHa and AI treatment, with limits of detection ranging from 10–20 pg/mL^12,13^. Prior studies demonstrate an increased risk of breast cancer events in postmenopausal patients on adjuvant AI not reaching sufficiently suppressed levels of E2 and estrone (E1)^14,15^. Failure to achieve complete estrogen suppression with GnRHa may also contribute to worse outcomes in premenopausal patients with HR-positive breast cancer. Thus, more data regarding the endocrine effects of GnRHa and clinical implications of this are urgently needed, particularly in younger women who stand to benefit the most from optimized OFS.

We sought to assess estrogen levels using validated ultrasensitive methods in premenopausal patients aged ≤ 40 years receiving GnRHa plus oral endocrine therapy for early and de novo metastatic HR-positive breast cancer at 1 and 4 years after diagnosis, determine predictive factors of incomplete E2 suppression, and explore the relationship between E2 levels during GnRHa treatment and survival outcomes.

## Results

### Study population

**Fig. 1:**
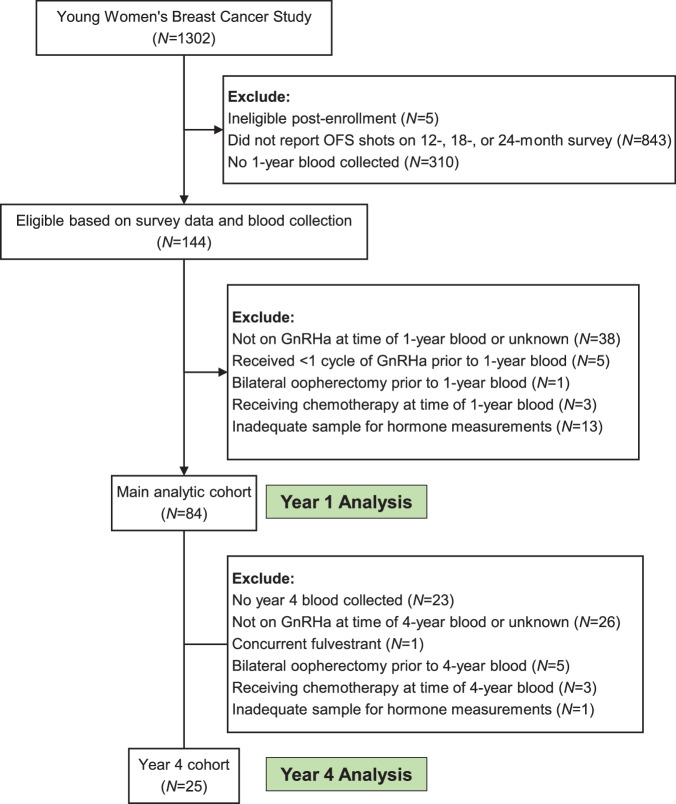
Study flow diagram. Abbreviations: OFS ovarian function suppression, GnRHa gonadotropin-releasing hormone agonist.

In total, 84 participants on GnRHa had a 1-year blood sample analyzed (Fig. 1). This group constituted the main analytic cohort (Table 1). At diagnosis, 10 patients (11.9%) were aged ≤ 30 years, 28 (33.3%) aged 31–35, and 46 (54.8%) aged 36–40, with a median age of 36 years (17–40 years). Median BMI was 24 kg/m^2^ (15–45 kg/m^2^), including 32% of patients BMI ≥ 25 kg/m^2^. The majority had stage I–III breast cancer (85.7%) and 12 (14.3%) had stage IV disease. GnRHa was administered monthly in 47 patients (56%) and every 3 months in 34 (40.5%), with leuprolide being the most frequently used agent (76.2%). Most patients were on concurrent tamoxifen (70.2%) and 25 were on AI (29.8%). A total of 49 patients (58.3%) had received prior chemotherapy.

**Table 1:** Patient characteristics in year 1 cohort, overall and according to occurrence of estradiol level > 2.72 pg/mL at 1 year

Characteristic	*N* (%)			*P*
	Overall (*N* = 84)	E2 ≤ 2.72 pg/mL (*N* = 38)	E2 > 2.72 pg/mL (*N* = 46)	
Age at diagnosis, years				
≤ 30	10 (11.9)	2 (5.3)	8 (17.4)	0.075
31–35	28 (33.3)	17 (44.7)	11 (23.9)	
36–40	46 (54.8)	19 (50.0)	27 (58.7)	
Race				
White	77 (91.6)	36 (94.7)	41 (89.1)	0.999
Asian	5 (6.0)	2 (5.3)	3 (6.5)	
Black	1 (1.2)	0 (0)	1 (2.2)	
Other	1 (1.2)	0 (0)	1 (2.2)	
BMI at diagnosis, kg/m^2^				
< 18.5	3 (3.6)	3 (7.9)	0	0.120
18.5–24.9	50 (59.5)	23 (60.5)	27 (58.7)	
≥ 25	27 (32.1)	10 (26.3)	17 (37.0)	
Missing	4 (4.8)	2 (5.3)	2 (4.3)	
Smoking history				0.158
Active/former	28 (33.3)	9 (23.7)	19 (41.3)	
Never	53 (63.1)	27 (71.0)	26 (56.5)	
Missing	3 (3.6)	2 (5.3)	1 (2.2)	
Alcohol history				0.448
Active	54 (64.3)	8 (21.0)	16 (34.8)	
Former	24 (28.6)	27 (71.1)	27 (58.7)	
Never	2 (2.4)	1 (2.6)	1 (2.2)	
Missing	4 (4.8)	2 (5.3)	2 (4.3)	
Stage				0.474
I	23 (27.4)	8 (21.0)	15 (32.6)	
II	38 (45.2)	18 (47.4)	20 (43.5)	
III	11 (13.1)	7 (18.4)	4 (8.7)	
IV	12 (14.3)	5 (13.2)	7 (15.2)	
HER2 status				0.277
Negative	67 (79.8)	28 (73.7)	39 (84.8)	
Positive	17 (20.2)	10 (26.3)	7 (15.2)	
Prior chemotherapy				0.045
No	35 (41.7)	11 (29.0)	24 (52.2)	
Yes	49 (58.3)	27 (71.1)	22 (47.8)	
Chemotherapy regimen*				0.152
Anthracycline plus taxane	29 (59.2)	17 (63.0)	12(54.5)	
Anthracycline-based	6 (12.2)	5 (18.5)	1 (4.5)	
Taxane-based	14 (29.0)	5 (18.5)	9 (40.9)	
GnRHa drug				0.174
Leuprolide	64 (76.2)	28 (73.7)	36 (78.3)	
Goserelin	14 (16.7)	5 (13.2)	9 (19.6)	
Triptorelin	6 (7.1)	5 (13.2)	1 (2.2)	
GNRHa schedule				0.485
Every month	47 (56.0)	23 (60.5)	24 (52.2)	
Every 3 months	34 (40.5)	13 (34.2)	21 (45.6)	
Unknown	3 (3.6)	2 (5.3)	1 (2.2)	
Endocrine therapy				0.009
Tamoxifen	59 (70.2)	21 (55.3)	38 (82.6)	
Aromatase inhibitor	25 (29.8)	17 (44.7)	8 (17.4)	

*± Trastuzumab ± pertuzumab in patients with HER2-positive breast cancer.

*E2* estradiol, *BMI* body mass index, *HER2* human epidermal growth factor 2 receptor, *GnRHa* gonadotropin-releasing hormone agonist.

The 4-year blood sample was analyzed in 25 participants (Supplementary Table 1). When compared to year 1, a similar proportion of patients were receiving monthly GnRHa (56%) versus every 3 months (44%) dosing, but a larger proportion of patients were on concurrent AI (44%).

### Estrogen and FSH levels at 1 and 4 years

**Fig. 2:**
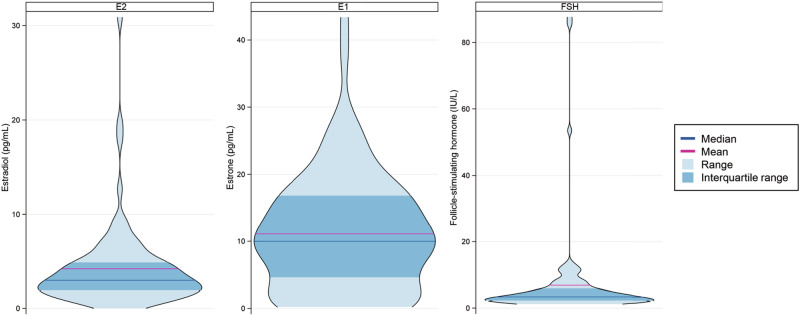
Violin plots of estradiol (E2), estrone (E1), and FSH levels at 1 year (*N* = 84).

At 1 year, median E2 for the overall study population was 3.0 pg/mL ( < 0.2–30.9 pg/mL, Table 2). E2 levels were > 10 pg/mL in four patients, including three on tamoxifen (75%) and one on AI (25%). The majority of patients had suppressed FSH levels, whereas E1 levels were more widely distributed (Fig. 2). Median time from GnRHa initiation to 1-year timepoint was 7 months (1–18 months). Among patients who received chemotherapy, median time from chemotherapy completion to 1-year timepoint was 8 months (2–20 months).

**Table 2:** Estradiol, estrone, and FSH levels at 1 year, overall and according to oral endocrine therapy

Hormone	Median (range)			*P*
	Overall (*N* = 84)	Tamoxifen (*N* = 59)	AI (*N* = 25)	
E2 (pg/mL)	3.0 ( < 0.2*–30.9)	3.9 (1.0–19.6)	2.1 ( < 0.2*–30.9)	0.001
E1 (pg/mL)	10.0 ( < 0.2*–43.4)	13.3 (0.2*–43.4)	1.4 ( < 0.2*–29.0)	< 0.001
FSH (IU/L)	3.3 (1.2–87.7)	2.5 (1.2–84.4)	7.1 (2.4–87.7)	< 0.001

*Estrogen concentrations below lower limit of quantification ( < 1 pg/mL) for assay have an accuracy of ± 50%.

*E2* estradiol, *E1* estrone, *FSH* follicle stimulating hormone.

At 4 years, median E2 was 3.7 pg/mL (1.0–18.8 pg/mL, Supplementary Table 2). E2 levels were > 10 pg/mL in 2 patients, including one on tamoxifen and one on AI.

There was a positive correlation between E2 and E1 levels at 1 year (*r* = 0.57, *P* < 0.001) and 4 years (*r* = 0.89, *P* < 0.001). E2 and FSH levels were not significantly correlated at 1 year (*r* = −0.15, *P* = 0.171), but were negatively correlated at 4 years (*r* = −0.55, *P* = 0.005). At both 1 and 4 years, median E2 and E1 levels were significantly higher among patients on concurrent tamoxifen compared to those on AI (Table 2, Supplementary Table 2). Median FSH levels were significantly higher in patients on AI than tamoxifen at 1 and 4 years.

### Patient characteristics and outcomes according to estrogen levels

In total, 46 patients (54.8%) had an E2 value > 2.72 pg/mL at 1 year. Patients with E2 levels > 2.72 pg/mL were less likely to have received prior chemotherapy (*P* = 0.045) and more likely to be on concurrent tamoxifen (*P* = 0.009) than patients whose E2 levels remained ≤ 2.72 pg/mL (Table 1). Thirty-two percent of patients on AI (8/25) and 65% of patients on tamoxifen (38/59) had E2 > 2.72 pg/mL. Eight of 10 patients (80%) aged ≤ 30 years had E2 > 2.72 pg/mL, compared to 39% of patients aged 31–35 years and 59% of patients aged 36–40 years, although this difference was not statistically significant (*P* = 0.075). BMI, tumor stage, GnRHa drug, and GnRHa schedule were not associated with E2 levels > 2.72 pg/mL.

At 4 years, 15 patients (60%) had an E2 value > 2.72 pg/mL. Patients with E2 levels > 2.72 pg/mL were more likely to be receiving GnRHa every three months (*P* = 0.012), leuprolide as the GnRHa drug (*P* = 0.004), and concurrent tamoxifen (*P* < 0.001). (Supplementary Table 1). In total, 10 patients had E2 levels > 2.72 pg/mL at both 1 and 4 years after diagnosis.

**Fig. 3:**
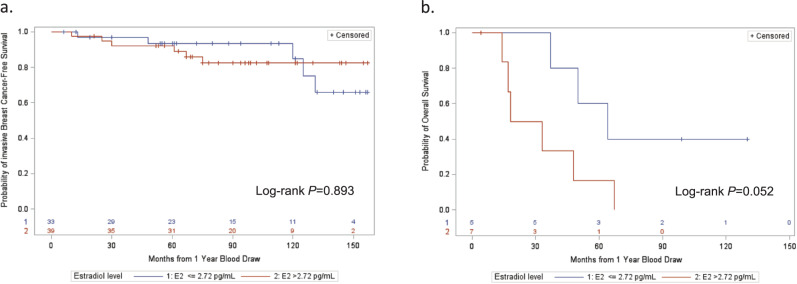
Kaplan-Meier curves for survival outcomes according to occurrence of estradiol level > 2.72 pg/mL at 1 year. **a** Invasive breast cancer-free survival in patients with stage I–III breast cancer (*N* = 72), and (**b**) overall survival in patients with de novo stage IV breast cancer (*N* = 12).

After a median follow-up of 7 years (1–13 years), 11 patients with stage I–III breast cancer had disease recurrence, including 4 locoregional and 7 distant recurrences, and 9 patients with stage IV breast cancer died. Among the 74 patients with early-stage breast cancer, iBCFS events were seen in 6 (8.1%) with E2 levels > 2.72 pg/mL and 5 (6.8%) with E2 ≤ 2.72 pg/mL at 1 year (*P* = 0.893, Fig. 3a). Among the 12 patients with stage IV breast cancer, OS events were seen in 6 (50%) with E2 > 2.72 pg/mL and 3 (25%) with E2 ≤ 2.72 pg/mL at 1 year (*P* = 0.052, Fig. 3b). Among the 22 patients with early-stage breast cancer on AI, iBCFS events were seen in 1 (4.5%) with E2 levels >2.72 pg/mL and 2 (9.1%) with E2 ≤ 2.72 pg/mL at 1 year (*P* = 0.442, Supplementary Fig. 1a). Among the 3 patients with stage IV breast cancer on AI, OS events were seen in 2 (66.7%) with E2 > 2.72 pg/mL and none with E2 ≤ 2.72 pg/mL (*P* = 0.225, Supplementary Fig. 1b).

## Discussion

OFS has become an important component of treatment for premenopausal patients with early and advanced HR-positive breast cancer. Yet unanswered questions remain regarding the optimal management of young women on pharmacologic OFS with GnRHa, including the role of E2 monitoring and clinical implications of detectable levels. Ensuring adequate OFS with GnRHa is likely to become an even greater issue as large multicenter trials investigating chemotherapy-sparing approaches in premenopausal patients with intermediate genomic risk HR-positive tumors are launched, including the planned NRG BR009 study^16^. Thus, this study provides timely data regarding the endocrine effects of GnRHa treatment in patients aged ≤ 40 years, with 55% of women having E2 levels greater than the ultra-low threshold of 2.72 pg/mL when measured 1 year after diagnosis. In contrast, 5% of patients had E2 levels greater than the conventional ovarian function breakthrough cut-off of 10 pg/mL. This suggests that, while GnRHa effectively lowers E2 levels in the majority of young women, complete E2 suppression is challenging for some in this population^1–3^.

Data on estrogen levels during GnRHa plus oral endocrine therapy in premenopausal breast cancer patients are limited, in particular by the use of inadequate analytical techniques that lack sensitivity and specificity to detect low hormone concentrations^17–23^. LC-MS/MS is a reliable method for quantitative E2 assessment over a wide range of physiological concentrations, including in postmenopausal patients on AI, in whom estrogen levels can reach below 1 pmol/L^24^. In addition to improved precision, LC-MS/MS is also highly selective compared to conventional immunoassays, which are often compromised by cross-reactivity with similar steroid-like compounds, such as exemestane^25,26^. While gas chromatography tandem mass spectrometry (GC-MS/MS) is often used as the reference method for ultrasensitive E2 measurement, LC-MS/MS offers advantages over GC-MS/MS, including smaller sample volume requirements, simpler sample preparation, and faster run times that improve throughput^25^, demonstrating its potential for routine clinical applications.

Apart from the present study, the SOFT-EST substudy is one of the few others that has measured E2 using ultrasensitive methods in premenopausal breast cancer patients receiving GnRHa and oral endocrine therapy^7^. In this study, 34% of patients on triptorelin plus exemestane had at least one E2 level > 2.72 pg/mL during the first 12 months of treatment. Similar to the present study, this finding was more frequent in patients who were younger and chemotherapy-naïve. SOFT-EST also found an association between high BMI and E2 levels > 2.72 pg/mL, in keeping with prior data in postmenopausal patients treated with AI, significantly correlating higher BMI with higher E2 levels^27^. The lack of association between BMI and E2 levels in the present study may be due to the lower proportion of patients who were on AI compared to SOFT-EST, lessening the potential impacts of obesity, and consequent increased total body aromatization, on the efficacy of AIs to fully inhibit the aromatase enzyme and suppress estrogen production^28^.

Not surprisingly, patients on concurrent tamoxifen were more likely to have E2 levels > 2.72 pg/mL on GnRHa treatment than those on AI, given the synergistic E2-suppressive effects of AI plus GnRHa in premenopausal women^29^. In contrast, the mixed agonist and antagonist effects of tamoxifen can paradoxically lead to increases in plasma E2 levels in premenopausal women^30–35^. Lowering of tamoxifen-induced E2 elevations by GnRH may in part explain the improved efficacy of tamoxifen plus GnRHa over tamoxifen monotherapy in this population^2,3^. Nevertheless, given the mechanism of action of tamoxifen, the value of E2 monitoring in patients receiving this drug is less clear compared to those receiving AI. It is notable, however, that 36% of patients in this study were able to achieve E2 levels ≤ 2.72 pg/mL on tamoxifen plus GnRHa, demonstrating that profound E2 suppression from GnRHa is possible even on tamoxifen.

Although E2 is the most potent estrogen and main estrogen product synthesized in premenopausal ovaries, E1 is the main estrogen product of aromatization and is produced in greater quantities during the postmenopausal period^36^. The mechanism of E1 biosynthesis would explain the considerable difference in median E1 levels observed between patients on tamoxifen (13.3 pg/mL) versus AI (1.4 pg/mL), and why patients on AI had comparatively higher median E2 (2.1 pg/mL) than E1 levels (1.4 pg/mL). Median FSH levels were appropriately low in both groups secondary to the direct suppressive effects of GnRHa; however, FSH levels were found to be significantly higher in patients on AI compared to tamoxifen. This likely reflects a release in E2 negative feedback on pituitary FSH secretion secondary to AI-mediated inhibition of peripheral estrogen synthesis^37^.

Prior studies investigating estrogen suppression with GnRHa plus oral endocrine therapies have also been limited by small numbers of young patients^19,21,22,38–45^, short follow-up^20–22,41–45^, and lack of correlation between E2 levels and clinical outcomes. The present study is unique in that it included a relatively large number of patients aged ≤ 40 years, a subgroup of great interest, given these patients appear to benefit the most from OFS^1^, yet are thought to have the highest risk of ovarian breakthrough. Median age of the study population was 36 years, compared to 44 years in SOFT-EST, which may explain why a greater proportion of patients on AI at 1 year had E2 levels > 2.72 pg/mL (32%) than in SOFT-EST (17%)^7^. The proportion of patients with E2 levels greater than 2.72 pg/mL remained high at 4 years post-diagnosis (60%), suggesting that more sensitive E2 monitoring in young women may be important during the first year of GnRHa treatment and beyond. Patient characteristics associated with E2 levels > 2.72 pg/mL differed somewhat at 4 years compared to 1 year, including receipt of GnRHa every 3 months. GnRHa adherence was not assessed in this study; and thus, whether this finding relates to waning efficacy or poorer compliance in the setting of lengthier treatment intervals cannot be determined. While prior studies have shown no significant difference in clinical or biochemical efficacy according to GnRHa schedule^46–48^, many still favor monthly GnRHa dosing in younger patients.

The clinical relevance of our findings and those of previous studies including SOFT-EST are unclear. In the present study, there were no statistically significant differences in iBCFS or OS observed between patients with and without E2 levels > 2.72 pg/mL. However, it is notable that patients with stage IV breast cancer and E2 > 2.72 pg/mL 1 year post-diagnosis had double the number of OS events compared to those with E2 ≤ 2.72 pg/mL, which bordered on statistical difference. An updated analysis of the SOFT-EST substudy similarly did not find a detrimental impact of E2 levels > 2.72 pg/mL on disease-free survival (DFS), with no DFS events occurring after a median follow-up of 6 years among the 21 patients with E2 levels > 2.72 pg/mL at two or more timepoints; however, the analysis was limited by few DFS events overall^49^. In contrast, there are some data in postmenopausal women to suggest that detectable estrogen levels during AI treatment, when measured via ultrasensitive assays, could increase the risk of disease recurrence. Using LC-MS/MS methods, Ingle et al. measured E2 and E1 levels after 6 months of adjuvant AI in a matched case-control study of postmenopausal patients with early-stage breast cancer^15^. Patients with E2 ≥ 0.5 pg/mL and E1 ≥ 1.3 pg/mL had a 2.25-fold (95% CI 1.42–3.47, *P* = 0.0006) increase in the risk of an early breast cancer event relative to patients with E1 and/or E2 below these thresholds after 6 months of AI treatment.

While other studies have also used ultralow E2 thresholds ( > 2.18 and > 2.72 pg/mL) to define ovarian function recovery on AI^9,10^, the optimal E2 cutoff for both postmenopausal patients on AI and premenopausal patients on GnRHa with AI or tamoxifen remains unknown. In clinical practice, E2 levels ≥ 10–40 pg/mL are commonly used to define premenopausal status and whether stricter E2 cutoffs impact clinical outcomes requires ongoing study^13^. In addition, limited accessibility to ultrasensitive estrogen assays can render measurement and interpretation of E2 levels challenging in clinical practice, increasing the potential for erroneous treatment decisions^12^. There is also considerable uncertainty about how best to manage elevated E2 levels when they occur in premenopausal patients during GnRHa treatment, with strategies ranging from adjusting the dose or interval between GnRHa injections, to elective bilateral oophorectomy. Novel pharmacologic OFS strategies, such as GnRH antagonists, are also being explored^50^.

This study should be interpreted in the context of its limitations. Though this study comprised a larger proportion of patients aged ≤ 40 years compared to SOFT-EST, the overall sample size was small and limited the ability to develop a model to evaluate predictive factors for E2 levels > 2.72 pg/mL during GnRHa treatment. The study was also underpowered to evaluate associations between E2 levels and survival outcomes, but nevertheless, provides preliminary data addressing this question. Reflective of the period of YWS study enrollment, most patients were on concurrent tamoxifen, where E2 thresholds consistent with adequate OFS from GnRHa are less clearly delineated. In the absence of a widely accepted definition, an E2 cutoff of 2.72 pg/mL was explored for all patients in the study, along with the more conventional cutoff of 10 pg/mL. While the prospective nature of the YWS cohort allowed for longer follow-up and inclusion of E2 measurements at both 1 and 4 years, serial E2 monitoring was not performed and the clinical significance of isolated elevations in E2 remains uncertain. However, 12% of patients did have E2 levels > 2.72 pg/mL at both timepoints, which could have a more unfavorable prognostic impact. The timing of blood collections varied somewhat from the intended 1- and 4-year timepoints from diagnosis among patients in this observational study, as did the timing of GnRHa initiation in relation to blood draw and chemotherapy, with provider preference dictating whether patients started GnRHa before or after chemotherapy if chemotherapy was received. Further, some factors that could influence E2 levels during GnRHa treatment were not evaluated, including symptoms (e.g., vaginal bleeding, cycling) and endocrine therapy adherence. Finally, our exclusion of women who had bilateral oophorectomy may have led to an underestimate of OFS breakthrough risk in very young women, given both the use of risk-reducing salpingo-oophorectomy in carriers of *BRCA1*/2 pathogenic variants, and that some who experienced symptoms or signs of OFS breakthrough likely underwent bilateral oophorectomy for management.

Nevertheless, over half of patients aged ≤ 40 years receiving GnRHa plus oral endocrine therapy for HR-positive breast cancer had at least one E2 level > 2.72 pg/mL during the first 4 years after diagnosis. Though these ovarian function breakthrough events did not impact survival outcomes in this relatively small sample, given the frequency of this phenomenon, larger studies are needed to elucidate optimal E2 levels for premenopausal women on GnRHa, and clinical implications of incomplete E2 suppression. As GnRHa use continues to increase and even replace adjuvant chemotherapy for some young patients, strategies to optimize OFS, including greater accessibility to validated ultrasensitive E2 assays, are imperative and could potentially improve outcomes for young women with this common subtype of breast cancer.

## Methods

### Study design

The Young Women’s Breast Cancer Study (YWS) is a multicenter, prospective cohort of women diagnosed with breast cancer at ≤ 40 years of age, enrolled between 2006 and 2016 from 13 sites in the United States and Canada. Women with newly diagnosed stage 0-IV breast cancer and able to respond to questionnaires in English were eligible. Potential participants at Dana-Farber/Harvard Cancer Center (DF/HCC) sites were identified by pathology review and elsewhere through a systematic review of clinic lists. Participants provided written informed consent authorizing medical record review, blood and tissue collection, and participant questionnaires. The YWS was conducted in accordance with all relevant ethical regulations including the Declaration of Helsinki and is approved by the institutional review board at DF/HCC and other participating centers.

### Study population

This analysis included participants with stage I–IV estrogen receptor (ER) and/or progesterone receptor (PR)-positive breast cancer who reported receiving OFS shots on the 12-, 24-, or 48-month follow-up survey and provided blood samples 1 year after diagnosis (median time from diagnosis to 1-year blood draw: 12 months [7–24 months], Fig. 1). Self-reported GnRHa receipt was confirmed by medical record review, with exclusion of participants determined not to be on GnRHa or to have received less than one cycle (defined as < 28 days if receiving monthly or < 84 days if receiving every 3 months) at time of 1-year blood draw. Participants on active chemotherapy, status post bilateral oophorectomy, or with inadequate blood samples for hormone assays were also excluded.

For the year 4 analysis, participants included those in the year 1 cohort who also provided blood samples 4 years after diagnosis (median time from diagnosis to 4-year blood draw: 48 months [43–61 months], Fig. 1). Participants not on GnRHa at the time of 4-year blood draw, receiving concurrent fulvestrant or chemotherapy, status post bilateral oophorectomy, or with insufficient blood samples were excluded.

### Covariate ascertainment

Race, height, weight, and substance use were self-reported on baseline and 4-year surveys. Pathology reports were used to abstract disease stage and receptor status. Chemotherapy and endocrine therapy information was obtained from medical record review. Chemotherapy regimens were categorized based on the entirety of treatment received for (neo)adjuvant therapy in patients with stage I–III disease and most recent treatment received in those with stage IV disease. Receipt of trastuzumab or pertuzumab was collapsed within the chemotherapy regimen, given prior data finding no impact of trastuzumab on treatment-related amenorrhea^51^. Medical record review was used to determine vital status and confirm self-report of disease recurrence or new primary breast cancer.

### Hormone assays

Prospectively collected blood samples were spun and stored at −80 °C as plasma and whole blood. Two millimeters of plasma was sent from each eligible participant per timepoint analyzed to the Brigham Research Assay Core Laboratory (Boston, MA). To obtain a more complete estrogen profile, both E2 and E1 concentrations were measured using a validated liquid chromatography-tandem mass spectrometry (LC-MS/MS) method, certified by the Centers for Disease Control and Prevention’s (CDC) Hormone Standardization Program, as previously described^52^. The lower limit of quantification (LLQ) for E2 and E1 was 1 pg/mL, with a measurement range of 1–500 pg/mL for E2 and 1–250 pg/mL for E1. Within these ranges, the intra- and inter-assay coefficients of variation (CV) are < 5%. For the LC-MS/MS E2 assay, the mean bias for quality control samples provided by the CDC’s Hormone Standardization Program was 0.81 pg/mL for concentrations ≤ 20 pg/mL and 1.9% for concentrations > 20 pg/mL.

Follicle-stimulating hormone (FSH) levels were determined by chemiluminescence using the Access Immunoassay System analyzer (Beckman Coulter, Fullerton, CA). The measurement range was 0.2–200 mIU/mL, with an intra-assay CV of 3.1–4.3% and inter-assay CV of 4.3–5.6%.

### Study objectives

Our primary objective was to estimate the proportion of patients receiving GnRHa plus oral endocrine therapy with E2 levels > 2.72 pg/mL ( > 10 pmol/L) at 1 and 4 years after diagnosis. This threshold was based on the recommended guidelines by Smith et al. to define E2 levels inconsistent with postmenopausal status on AI^11^. Given there is no universally-accepted standard for determining adequate OFS while on GnRHa and tamoxifen, this threshold was explored for all patients regardless of oral endocrine therapy type. The proportion of patients with E2 levels > 10 pg/mL was also evaluated.

Secondary objectives included evaluation of patient characteristics associated with E2 levels > 2.72 pg/mL at both timepoints and assessment of survival endpoints in relation to 1-year E2 levels > 2.72 pg/mL. Invasive breast cancer-free survival (iBCFS), defined as the time from 1-year blood draw to locoregional invasive ipsilateral recurrence, contralateral invasive breast cancer, distant recurrence, or death from any cause, was calculated in patients with stage I–III breast cancer. Overall survival (OS), defined as the time from 1-year blood draw to death from any cause, was calculated in patients with stage IV breast cancer. Patients without an event of interest were censored at the date of last follow-up.

### Statistical analysis

Patient characteristics potentially related to hormone levels were determined a priori and summarized as counts and proportions. Levels were summarized as medians with ranges, with Wilcoxon rank sum test used to compare values between patients on tamoxifen versus AI. E2 and E1 concentrations below the limit of detection (LOD) were inputted at the LOD. The relationship between hormone levels was assessed using Pearson correlation. Patient characteristics were compared for those with E2 levels > 2.72 pg/mL versus ≤ 2.72 pg/mL at 1 and 4 years using Wilcoxon rank sum and Fisher’s exact tests. Survival analyses were performed using Kaplan–Meier methods, with log-rank tests to compare patients with E2 levels > 2.72 pg/mL and ≤ 2.72 pg/mL at 1 year. Exploratory survival analyses including only those patients on AI were also carried out. All *p*-values were 2-sided and values less than 0.05 were considered statistically significant. Analyses were conducted using SAS 9.4 and StataBE 17.0.

### Supplementary information

Supplemental Material

## Data Availability

The datasets used and/or analyzed during the current study are available from the corresponding author on reasonable request.

## Code availability

The underlying code for this study is not publicly available but may be made available to qualified researchers on reasonable request.
