# Supplementary material for: Estrogen levels in young women with hormone receptor-positive breast cancer on ovarian function suppression therapy
Source: NPJ Breast Cancer. 2024 Aug 1;10:67. doi: 10.1038/s41523-024-00680-0 (PMC11294545; doi:10.1038/s41523-024-00680-0)
Supplement: Supplementary file 1 — Supplemental Material [file 41523_2024_680_MOESM1_ESM.pdf]

Supplementary Material

Estrogen levels in young women with hormone receptor-positive breast cancer on ovarian function suppression with gonadotropin-releasing hormone agonists

Supplementary Figures: 1

Supplementary Tables: 2

Supplementary Figure 1: Kaplan-Meier curves for survival outcomes according to occurrence of estradiol level >2.72 pg/mL at 1 year: a) invasive breast cancer-free survival in patients with early-stage breast cancer on an aromatase inhibitor (*N*=22), and b) overall survival in patients with stage IV breast cancer on an aromatase inhibitor (*N*=3).

a.

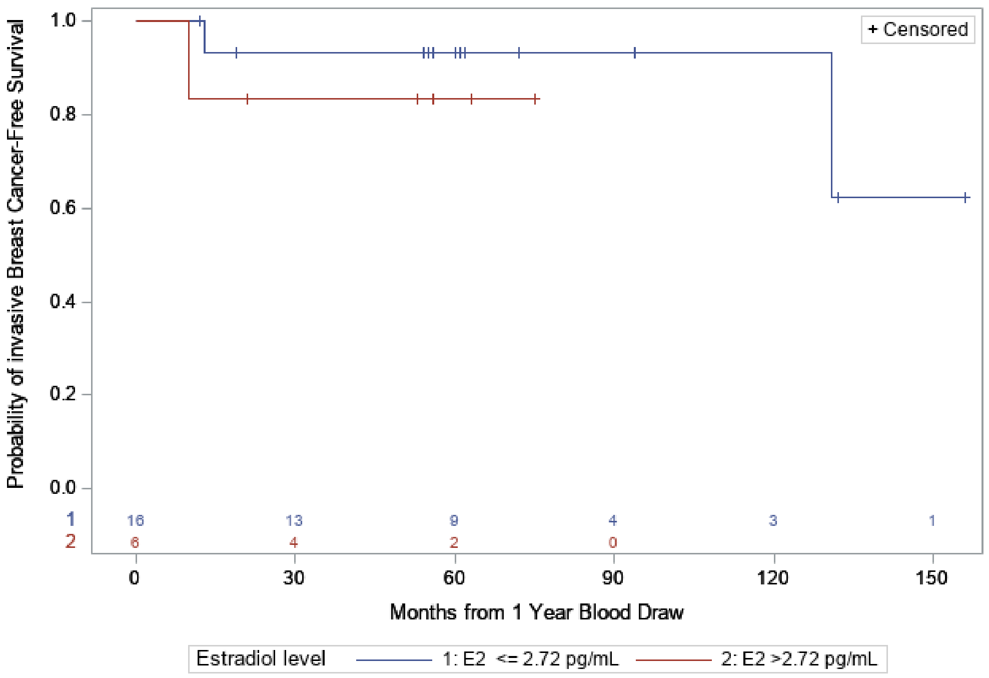

b.

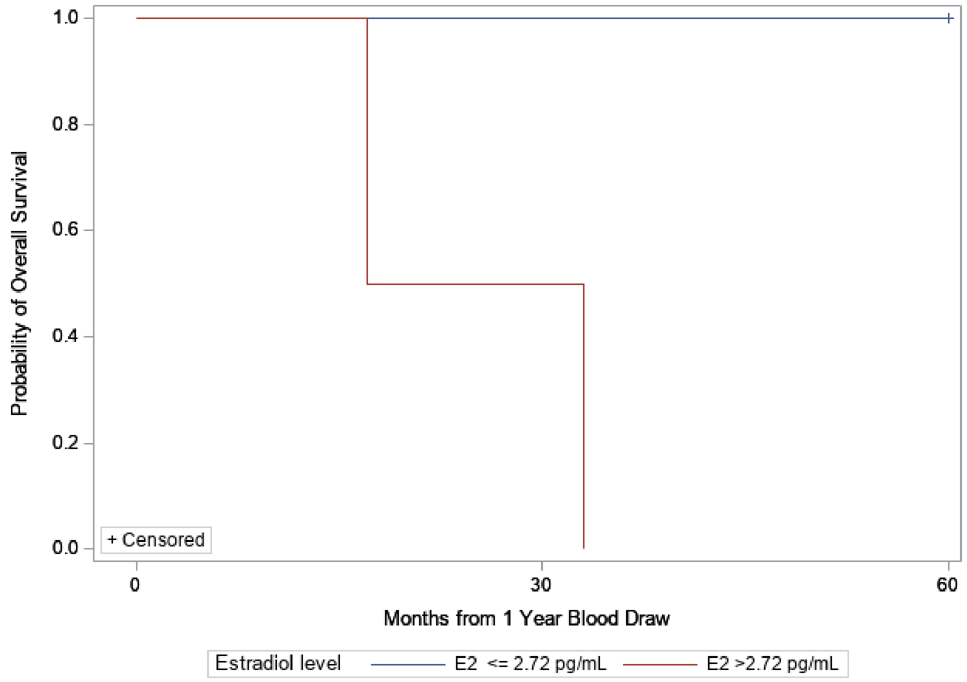

Supplementary Table 1: Patient characteristics in year 4 cohort, overall and according to occurrence of estradiol level >2.72 pg/mL at 4 years.

Characteristic	Overall (*N*=25)	*N* (%) E2 ≤2.72 pg/mL (*N*=10)	E2 >2.72 pg/mL (*N*=15)	*P*
Age at diagnosis, years				
≤ 30	2 (8.0)	0	2 (13.3)	0.487
31-35	9 (36.0)	5 (50.0)	4 (26.7)	
36-40	14 (56.0)	5 (50.0)	9 (60.0)	
Race				>0.999
White	24 (96.0)	10 (100.0)	14 (93.3)	
Asian	1 (4.0)	0	1 (6.7)	
BMI at year 4, kg/m^2				0.796
<18.5	1 (4.0)	0	1 (6.7)	
18.5-24.9	10 (40.0)	5 (50.0)	5 (33.3)	
≥ 25	12 (48.0)	4 (40.0)	8 (53.3)	
Missing	2 (8.0)	1 (10.0)	1 (6.7)	
Smoking history at year 4				0.657
Active/former	8 (4.0)	2 (20.)	6 (40.0)	
Never	16 (64.0)	7 (70.0)	9 (60.0)	
Missing	1 (32.0)	1 (10.)	0	
Alcohol history at year 4				>0.999
Active	23 (92.0%)	10 (100.)	13 (86.7)	
Former	1 (4.0%)	0	1 (6.7)	
Missing	1 (4.0%)	0	1 (6.7)	
Stage				0.214
I	7 (28.0)	1 (10.0)	6 (40.0)	
II	13 (52.0)	6 (60.0)	7 (46.7)	
III	4 (16.0)	2 (20.0)	2 (13.3)	
IV	1 (4.0)	1 (10.0)	0	
HER2 status				0.064
Negative	20 (80.0)	6 (60.0)	14 (93.3)	
Positive	5 (20.0)	4 (40.0)	1 (6.7)	
Prior chemotherapy				0.659
No	7 (28.0)	2 (20.0)	5 (33.3)	
Yes	18 (72.0)	8 (80.0)	10 (66.7)	
Chemotherapy regimen*				0.257
Anthracycline plus taxane	9 (50.0)	4 (50.0)	5 (50.0)	
Anthracycline-based	3 (16.7)	0	3 (30.0)	
Taxane-based	6 (33.3)	4 (40.0)	2 (20.0)	
GnRHa drug				0.004
Leuprolide	17 (68.0)	5 (50.0)	12 (80.0)	
Goserelin	5 (5.0)	5 (50.0)	0	
Triptorelin	3 (12.0)	0	3 (20.0)	

GNRHa schedule				0.012
Every month	14 (56.0)	9 (90.0)	5 (33.3)	
Every 3 months	11 (44.0)	1 (10.0)	10 (66.7)	
Endocrine therapy				<0.001
Tamoxifen	14 (56.0)	0	14 (93.3)	
Aromatase inhibitor	11 (44.0)	10 (100.0)	1 (6.7)	

*±Trastuzumab ±pertuzumab in patients with HER2-positive breast cancer.

Abbreviations: E2, estradiol; BMI, body mass index; HER2, human epidermal growth factor 2 receptor; GnRHa, gonadotropin-releasing hormone agonist

Supplementary Table 2: Estradiol, estrone, and FSH levels at 4 years, overall and according to oral endocrine therapy.

**Hormone**	**Median (range)**			***P***
	**Overall (*N*=25)**	**Tamoxifen (*N*=14)**	**AI (*N*=11)**	
E2 (pg/mL)	3.7 (1.0*-18.8)	4.7 (2.9-10.4)	2.0 (1.0*-18.8)	<0.001
E1 (pg/mL)	6.2 (<0.2*-33.1)	11.5 (3.2-25.0)	1.4 (0.1*-33.1)	<0.001
FSH (IU/L)	1.9 (1.1-9.3)	1.4 (1.1-2.5)	6.2 (1.8-9.3)	<0.001

*Estrogen concentrations below lower limit of quantification (<1 pg/mL) for assay have an accuracy of ±50%.

Abbreviations: E2, estradiol; E1, estrone; FSH, follicle stimulating hormone
